# Medical law; promotion of medicine curriculum: a letter to editor

**DOI:** 10.1080/10872981.2023.2290333

**Published:** 2023-12-05

**Authors:** Bahar Moasses Ghafari, Taraneh Khodaparast, Parsa Hasanabadi

**Affiliations:** a Medicine Faculty, Kurdistan University of Medical Sciences, Sanandaj, Iran; b Department of Law, Islamic Azad University, Sanandaj branch, Sanandaj, Iran; c Student Committee of Medical Education Development, Education Development Center, Kurdistan University of Medical sciences, Sanandaj, Iran; d Student, Medicine Faculty, Kurdistan University of Medical Sciences, Sanandaj, Iran

Dear editor;

According to ever-increasing advances, doctors and medical students face numerous challenges across various fields. Among these challenges, the rights of doctors, students, and patients are of utmost importance. Due to civil law, each party (doctors, students, and patients) has specific rights that should be defined in society, the medical system, and medical education. It is crucial to determine the legal limits for these individuals. Therefore, it becomes imperative to establish the legal boundaries for these individuals, necessitating changes in the medical students’ education, with an expanded curriculum serving as the initial step. Consequently, for the following reasons, this innovative educational program should be expanded in medical schools; this curriculum expansion is based on the preparation of national medical textbooks by subject, taking into account the local cultural and religious conditions along with integration of the judicial laws of the region related to possible foreseeable issues facing medical doctors under the supervision of the Ministry of health of each country, is suggested as a primary practical solution [1].

Historically, the medical profession has been subject to numerous complaints. In 2020, just during the COVID-19 outbreak, when the number of visits to medical service centers increased, health services, including medicine, were recognized as one of the 10 job groups that received the most complaints [2]. As medical students someday work as doctors, their awareness of their rights and those of patients can significantly improve service efficiency, reduce errors, and minimize complications. Familiarity with the consequences of negligence or inadequate knowledge makes this educational program a preventive measure against learning deficiencies. Integrating this subject into the curriculum develops valuable and functional effects, facilitating early attention to common errors and challenges doctors face, improving student performance, and reducing probable errors [3]. Increased attention to errors, also enhances students’ scientific reasoning abilities.

Furthermore, considering that a significant number of errors made by doctors stem from a lack of effective communication and communication skills, ignorance of cognitive bias factors these issues can be mitigated in the long run. Additionally, supporting the MD/JD (Doctor of Medicine and Doctor of Jurisprudence) dual degree can benefit doctors. This dual degree equips individuals with medical and legal expertise, enabling them to excel in healthcare oversight, medical malpractice protection, health policy, medical ethics, and legal aspects of medical research. By combining medical and legal knowledge, professionals are better prepared to address complex legal issues in medical practice. They are ultimately donating to improved healthcare outcomes and patient safety. Therefore, this course and MD/JD synergistically affect the development of clinical reasoning, effective communication, and overall student training. Ultimately, these factors contribute to increased professionalism, trust in the judicial system, and the expansion of case-based learning [4]. Medical law also positively impacts teaching and learning by stimulating critical thinking and addressing biases that can lead to complications [5]. Familiarity with laws enables clinicians to differentiate between legal and illegal issues, making medical law a practical lesson. Combining medical law with ethics empowers educators to act more effectively in various situations and equips professionals to handle complex legal matters [6]. Patients also benefit from this curriculum, particularly in areas such as Quality of Care, Patient Safety, designing studies involving patient intervention, and medical services. Other parties that benefit from this course are the Ministry of Health, and organizations involved with medical errors that differ in distinct countries.

After knowing about the importance of medical law courses, a question arises regarding when this subject should be introduced during medical students’ training period? Should it be during general medicine or specialty training? In response to this question, we can refer to Zander Bratland et al.‘s paper that most complaints occur from general practitioners (without completing a specialized course) [5]. Therefore, it is suggested that this subject be presented during the internship period of these students and in residency programs as a continuing curriculum. Also, studies have shown that situated-learning theory (SLT) applied through court-based learning (CBL) is an effective learning method [4]. In this scenario, law students specializing in medical law could engage in CBL by observing and analyzing actual medical malpractice trials. Throughout this experience, students would apply the principles of SLT, wherein learning occurs within authentic contexts, to understand how medical law operates in real-world legal proceedings. Proficiency in legal matters enables clinicians to distinguish between legal and illegal issues, making medical law a pragmatic subject.

We hope this letter highlights the benefits of expanding the curriculum to enhance this important societal need
